# A mind–body intervention for stress reduction as an adjunct to an information session on stress management in university students

**DOI:** 10.1002/brb3.1651

**Published:** 2020-05-07

**Authors:** Mauro Cozzolino, Laura Girelli, Deborah R. Vivo, Pierpaolo Limone, Giovanna Celia

**Affiliations:** ^1^ Department of Humanities, Philosophy and Education University of Salerno Fisciano Italy; ^2^ Department of Humanities, Literature, Cultural Heritage, Education Sciences University of Foggia Foggia Italy

**Keywords:** brain wave modulation, mind–body therapies, organizational stress interventions, psychosocial stress, stress coping, stress management, university students

## Abstract

**Introduction:**

This study describes the implementation of a mind–body intervention to reduce the perceived level of stress in a nonclinical group of university students. We used a novel approach including a single session of a mind–body technique known as the brain wave modulation (BWM) as an adjunct to a single information session on stress management.

**Methods:**

Three hundred and six students participated in the study. A quasi‐experimental design was adopted: Students in the experimental group were exposed to an information session on stress management followed by a single session of the BWM, while the other students were exposed to the information session alone.

**Results:**

A 2 × 2 mixed factor analysis of variance demonstrated that the single session of the BWM was effective in reducing the perceived level of stress in the experimental group as compared to the control group.

**Conclusion:**

The BWM is a very easy‐to‐learn technique that presents certain advantages over traditional mind–body methods.

## Graphical abstract

This study describes the implementation of a mind–body intervention to reduce the perceived level of stress in a nonclinical group of university students. We used a novel approach including a single session of a mind–body technique known as the brain wave modulation (BWM) as an adjunct to a single information session on stress management. Results indicate that the single session of the BWM was effective in reducing the perceived level of stress in the experimental group as compared to the control group.

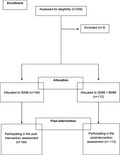

## 1 INTRODUCTION

Mind–body therapies can be defined as interventions that are based on several practices designed to facilitate the mind's positive impact on the body (American Academy of Pediatrics, 2016). Ancient practices for self‐care and well‐being such as meditation, yoga, tai chi, and qigong, as well as more modern western practices such as hypnotherapy, progressive relaxation, autogenic training, mindfulness, biofeedback, guided imagery, relaxation training, and psychological therapies, all embrace this definition. The list is ever growing and includes new practices developed over the past few decades that integrate psychology, consciousness, and body movement, such as eye movement desensitization reprocessing (Fernandez & Faretta, 2007), mind–body transformation therapy (Rossi, Mortimer, & Rossi, 2013), and brain wave modulation (Cozzolino & Celia, 2016), which we used for the purposes of this study in a population of university students.

University students experience significant stress throughout their studies as they struggle with several academic concerns, family problems, and issues relating to intimate and social relationships, finances, personal appearance, and personal health. They often report feeling overwhelmed, and increases in drug abuse, depression, anxiety, and suicidality have also been observed (Beiter et al., 2015; Liu, Stevens, Wong, Yasui, & Chen, 2019). Research associates excess stress with increases in physical symptoms such as headaches, sleep disturbances, cold hands and feet, neck and backaches, fatigue (Pozos‐Radillo, Preciado‐Serrano, Plascencia‐Campos, & Rayas‐Servín, 2015), and the common cold (Janicki Deverts, Cohen, & Doyle, 2017).

Recent studies demonstrated that stressful life events (SLE), defined as exposures to events which were perceived as somehow traumatic by the subjects (such as moving away from home or becoming ill), were high and associated with mental health issues, especially internalizing symptoms, that is., depressive and anxiety symptoms (Duprey, McKee, O’Neal, & Algoe, 2018; Liu et al., 2019). A recent line of research (Cozzolino, Guarino, Castiglione, Cicatelli, & Celia, 2017; Hsieh & Eisch, 2010; Lloyd & Rossi, 2008; Niles, Mehta, Corrigan, Bhasin, & Denninger, 2014; Rossi, Cozzolino, Mortimer, Atkinson, & Rossi, 2011; Rossi, Rossi, Yount, Cozzolino, & Iannotti, 2006) has demonstrated that the cause of many disease conditions, including inflammatory and neurodegenerative diseases, is a complex interaction among distressful life experiences, genome, mind, and behavioral factors. These studies provide new insights into the pathophysiology of stress‐related disorders and have identified the gene sets involved in several biological pathways, including stress response, inflammation, and physical health. They also describe the genomic and epigenetic pathways of stress focusing on gene expression changes brought about by mind–body therapies.

Other studies (Finkelstein‐Fox, Park, & Riley, 2018; Gallego, Aguilar‐Parra, Cangas, Rosado, & Langer, 2016; Meier & Welch, 2016; Running & Hildreth, 2017; Saoji, Mohanty, & Vinchurkar, 2017) supported the effectiveness of a number of mind–body interventions in reducing stress in university students. These interventions include bioenergy, biofeedback, mindfulness, autogenous training, cognitive‐behavioral therapy, yoga, and tai chi.

Despite a number of studies supporting the effectiveness of these interventions in lowering the level of stress, (Gallego et al., 2016; Nanthakumar, 2018; Stillwell, Vermeesch, & Scott, 2017; Upchurch, Gill, Jiang, Prelip, & Slusser, 2018; Wang & Hagins, 2016), improving well‐being (Birtwell, Williams, van Marwijk, Armitage, & Sheffield, 2019; Sarkissian, 2014; Soares & Chan, 2016; Zhang et al., 2018), and academic attainment in student populations (Bennett & Dorjee, 2016), they may also present adverse effects and contraindications. Notwithstanding the scarcity of empirical investigation on this subject, it is reported that mindfulness and meditation may induce temporary neurotic/anxiety symptoms (Dobkin, Irving, & Amar, 2012); mindful meditation, in particular, may induce psychosis, mania, or suicidal ideation especially in those with prior psychiatric morbidity (Wielgosz, Goldberg, Kral, Dunne, & Davidson, 2019); the autogenous training is contraindicated for those who suffer from a number of medical and/or mental conditions, for example, hypertension and psychosis (Brancaleone, 2010); yoga may not be recommended for those who suffer from arthritis (Dobkin et al., 2012); guided imagery may trigger post‐traumatic stress symptom in patients with a history of previous emotional, sexual, or physical abuse (American Academy of Pediatrics, 2016); and qigong is contraindicated for those who have suffered from mental illness or severe consumptive disease, cerebrovascular disease, and severe cardiovascular, liver, kidney, gastrointestinal and hematological diseases, and musculoskeletal system diseases (Guo et al., 2018). Clearly, exercise‐based mind–body techniques such as yoga, tai chi, and qigong are not suitable for those who are advised against mild to moderate physical exertion.

In addition, mind–body therapies often require a training that may be challenging to learn and are generally time‐consuming to perform. Other reasons why people eventually dropout of such programs include conflicting time demands and discomfort working in groups (Dobkin et al., 2012).

In this study, we chose a stress reduction method called the brain wave modulation (BWM), (Cozzolino & Celia, 2016; Hirai, 1975), in the conviction that it may determine a significant stress reduction in a population of students, while avoiding some of the issues posed by other methods.

The BWM is a stress reduction technique, which originates from the interaction between Eastern doctrines and the scientific study of the mind–body dialogue (Cozzolino & Celia, 2016). The rationale of the technique traces back to the studies led in the Seventies by Tomio Hirai, one of the most influential Japanese psychiatrists of that age. His experimental studies on nocturnal sleep and brain waves (Hirai, 1975; Hirai, Nomura, & Sekino, 1969; Hirai, Takano, & Uchinuma, 1968; Kasamatsu & Hirai, 1973) highlighted the extraordinary beneficial effects of the Zazen practice (seated meditation practiced by priests and disciples of Zen sects of Buddhism) on the human brain. In particular, Hirai (1975) demonstrated that Zen meditation induced the slowing of EEG patterns parallel with the disciples' mental states and specific changes in consciousness. Therefore, the BWM arises from the integration of Hirai's findings with recent mind–body procedures (Cozzolino & Celia, 2016; Rossi et al., 2011; Schriner, 1990) under a neuroscientific and clinical approach. The technique involves an easy‐to‐implement 4‐step finger movement procedure (see Appendix 1), and spontaneously helps our brain to release slower alpha waves (Desai, Tailor, & Bhatt, 2015).

The BWM presents certain advantages over traditional mind–body interventions. First, it is very easy to learn and can be performed in minutes. Therefore, it is a sustainable and reproducible intervention that students might prefer over other methods that are more time‐consuming and difficult to learn. Second, neither special premises nor specific equipment is required, so the BWM can be performed virtually everywhere. Third, the intervention can be administered individually as well as to a large number of subjects at the same time in a single session, as in this study, and it takes just one psychologist, which simplifies scheduling. Moreover, once the subjects have learned the technique, they can perform it autonomously at home. Finally, no side effects were reported by any of the participants in this study. We acknowledge that further research is needed into the possible contraindications and side effects that the technique might produce in clinical subjects with physical and/or mental conditions such as depression, cognitive deficiencies, PTSD, and TBI (Purohit et al., 2013), though in our clinical practice, we have already successfully used this technique in several patients with these conditions (Cozzolino & Celia, 2016).

In this study, we evaluated the effectiveness of the BWM as an adjunct to an information session on stress management in reducing part of the negative psychological perceived stress in a population of first‐year university students. In the first stage of the study, we conducted an information session on stress management in both the experimental and the control group. The information session on stress management was focused on providing the students with information about how to cope with stressful life events and preserve their mental health. It is generally acknowledged that developing a thorough understanding of the challenges, as well as a good knowledge of personal coping ability, helps individuals to address difficulties and feel more in control, which ultimately results in increased mental and emotional well‐being (van Daele, Hermans, van Audenhove, & van den Bergh, 2012).

Moreover, the study aimed at understanding whether a stress management program for university students based on the BWM as an adjunct to an information session on stress management would be effective and sustainable.

## 2 METHODS

### 2.1 Participants

**FIGURE 1:**
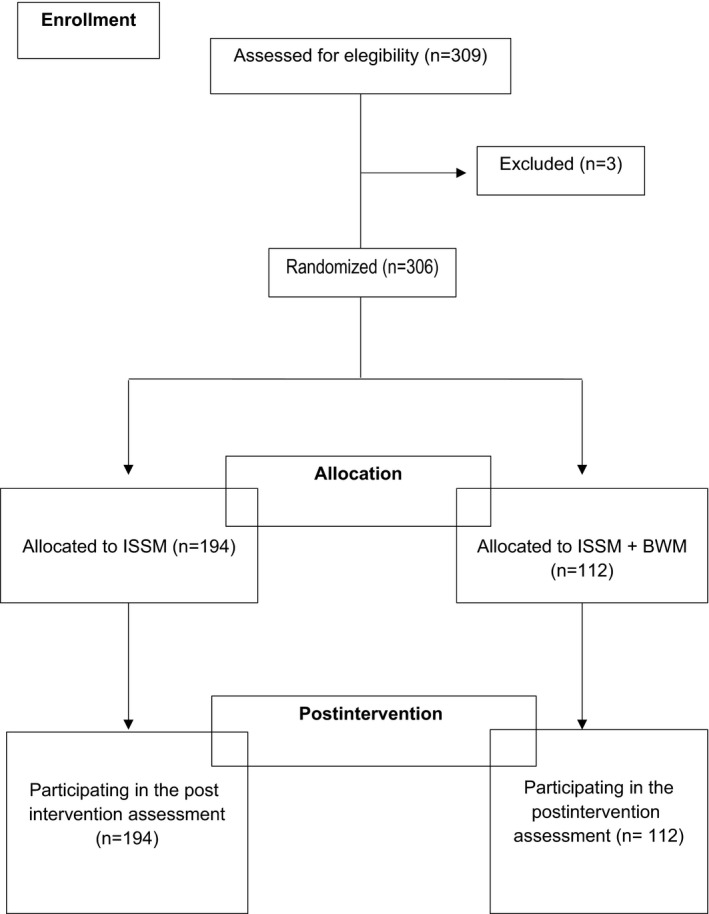
Flow diagram of the study

Participants were first‐year university students from the bachelor's degree in Sport Sciences, who attended psychology lessons. Three hundred and nine people had interest in taking part in the study. Exclusion criteria were age under 18 and severe mental or physical conditions. Three hundred and six students were eligible for the study. They were randomly allocated to the information session on stress management (ISSM) alone group condition (*n* = 194) or the ISSM + BWM group condition (*n* = 112). Of the *n* = 306 analyzed participants, 43,1% were women, mean age 20.5 years. The flow diagram of the study is displayed in Figure 1.

### 2.2 Materials and procedure

We used a quasi‐experimental design to evaluate the effect of a single mind–body intervention on a group of randomly selected first‐year university students. In order to perform randomization, we assigned students whose registration number ended with an odd number to the experimental group and the ones whose registration number ended with an even number to the control group. Since the student participation rate in previous experiments had been quite low, we hypothesized 30% of dropouts (*n* = 92), for a total of approx. 214 participants. The classroom we had requested for the experiment was the only one available on campus, which met requirements of light and quietness—needed for the technique to be performed in the best possible conditions. The experimental classroom, though, could comfortably accommodate only 112 people. Contrary to our expectations, there were no dropouts on the day of the experiment. Therefore, since we could not increase the experimental group, nor reject participants, we included more participants in the control group (*n* = 194).

Before the intervention started, the distress thermometer (DT, Jacobsen et al., 2005) was administered to all participants (pretreatment, time0). The DT is a screening tool that is well‐validated to be sensitive and specific to the construct of stress (Snowden et al., 2014). It includes a single item; therefore, subjects only have to indicate their perceived level of stress on a 1–100 scale. Thanks to its brevity, the DT is an ideal screening tool to include in a study on stress management with university students.

All participants received a 15‐min information session on stress management. Using video slides, the psychologist gave them information about stress, including how it acts, and how it produces negative effects on our body and our mind. After that, he went on presenting some stress coping strategies, such as progressive relaxation, autogenic training, and mindfulness.

After the information session on stress management was finished, the students in the experimental group stayed to receive a 15‐min single session of the brain wave modulation (BWM), (see Appendix 1), whereas the students in the control group were asked to kindly go wait in another room for 20 min.

**FIGURE 2:**
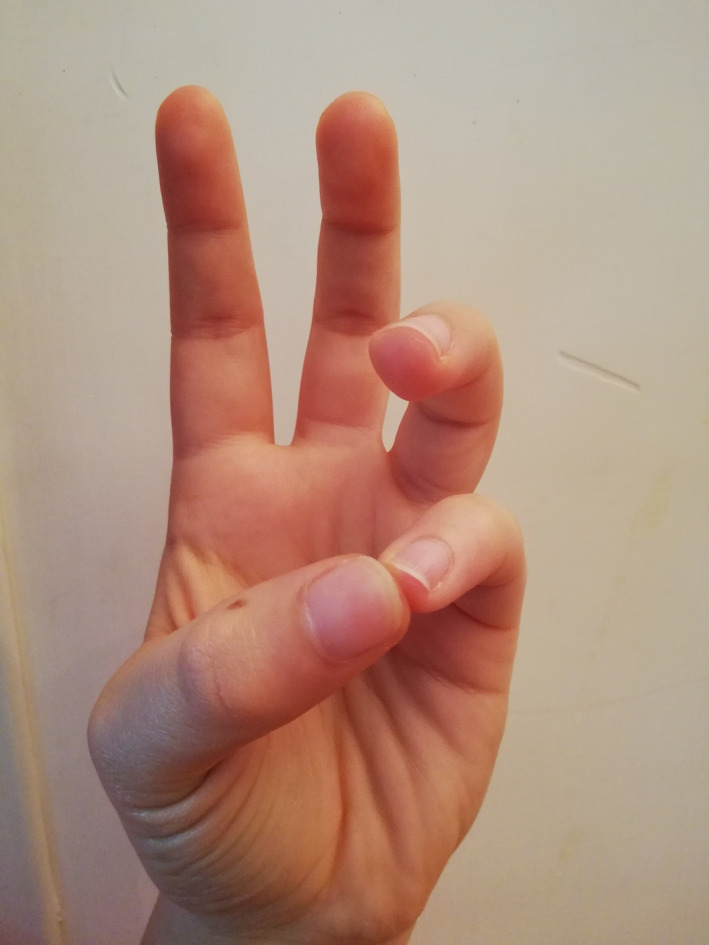
The first finger position of the BWM

**FIGURE 3:**
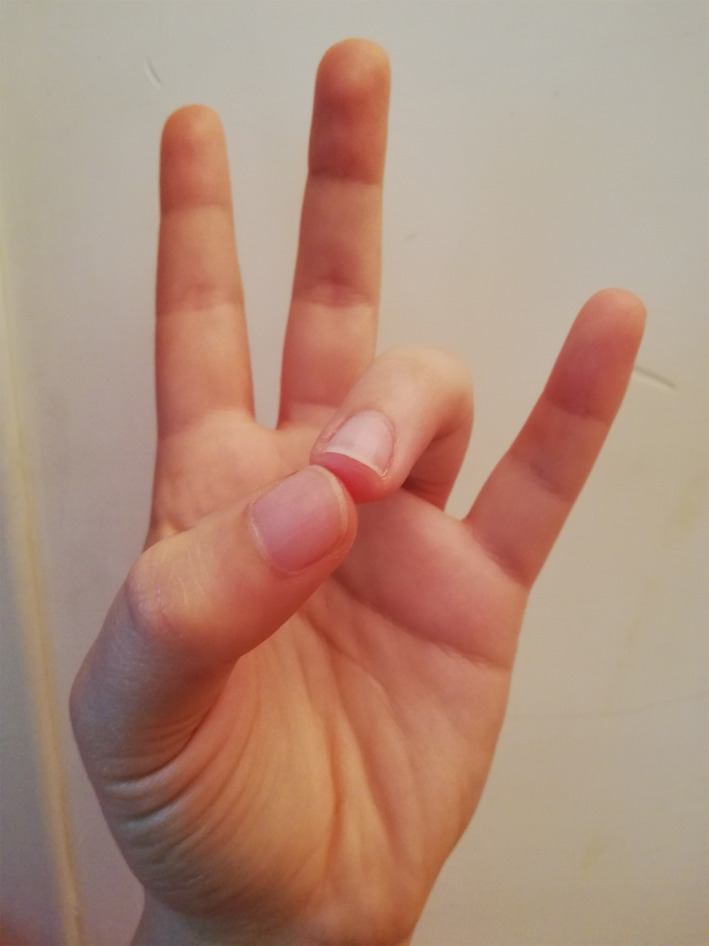
The second finger position of the BWM

**FIGURE 4:**
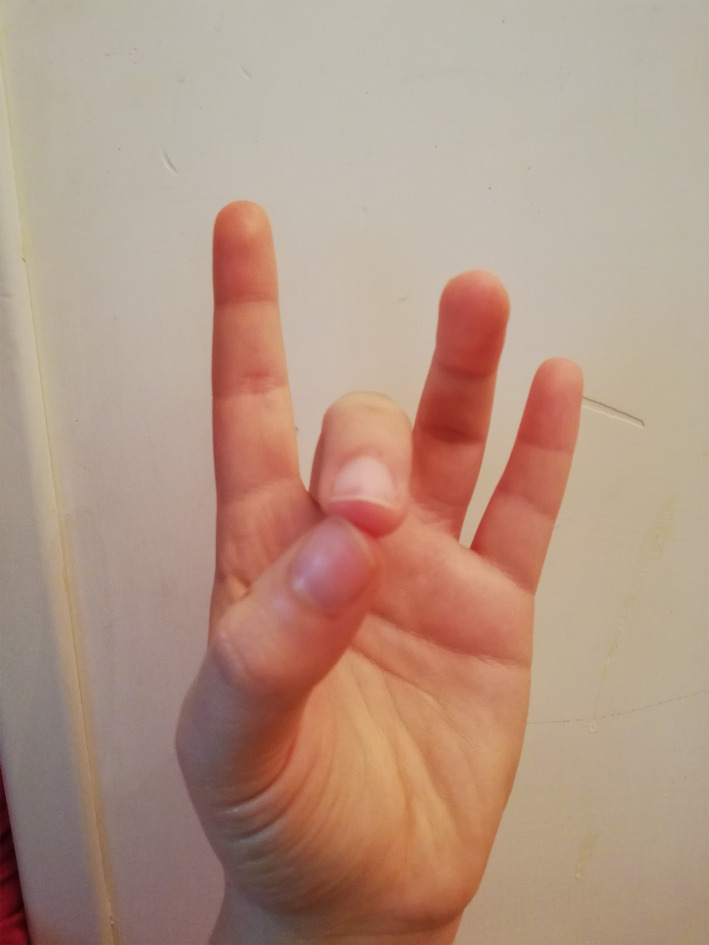
The third finger position of the BWM

**FIGURE 5:**
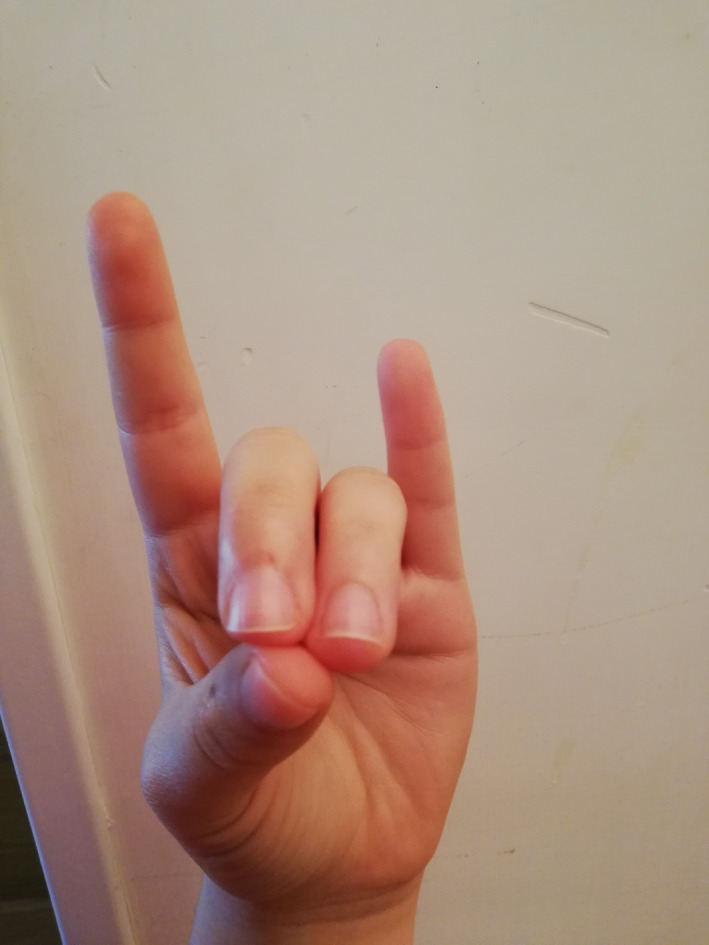
The fourth and last finger position of the BWM

The BWM technique used in the experimental group involves an easy‐to‐implement 4‐step finger movement procedure (see Appendix 1). Through the four positions, the physiology of our mind–body asset changes, gradually creating an activation of the parasympathetic system. The hemispherical dominance from left to right changes as well, which, in turn, automatically generates lowered pulse rate, lowered breathing rate, and muscle relaxation (Cozzolino & Celia, 2016; Hirai, 1975). In the first step already (Figure 2), the BWM naturally helps our brain to release slower alpha waves (Desai et al., 2015), characterized by a relaxed wakefulness that becomes even slower during the second step (Figure 3). In the third step (Figure 4), the technique produces typical sleep waves such as theta waves (Lagopoulos et al., 2009). Then, in the fourth step (Figure 5), the brain starts to produce delta waves, that is., the very deepest level of sleep and mental relaxation (Hondrou & Caridakis, 2012). Through these four steps, fast and intensive waves are replaced by slower and larger waves that are typical of relaxation and deep sleep (Cozzolino & Celia, 2016).

During the experiment, the psychologist introduced the technique and described it for about 5 min, showing each of the four BWM finger positions with his own hands, so that the students could mirror them and easily learn them. The psychologist then asked everyone to close their eyes and to start the session by putting their fingers in the first position shown earlier on. Then, the psychologist asked the students to use the other finger positions previously shown at intervals of three minutes. Three minutes after the last finger position, the psychologist asked the students to open their eyes. Overall, the intervention lasted about thirty minutes.

After the intervention was over, the psychologist asked the students how they were feeling and prompted them to indicate any unusual reactions they might have experienced in relation to the experiment. The participants did not report anything unusual after the intervention. However, the psychologist also gave his contacts to the students so that they could report any reactions relating to the experiment over the next few days. No such reports were received.

Once the postintervention discussion was finished, the DT (Jacobsen et al., 2005) was readministered to all participants (post‐treatment: time1). Informed consent from all participants was requested and obtained.

In order to evaluate the effectiveness of the BWM as an adjunct to an information session about stress management in reducing the perceived level of stress of a population of university students, a 2 × 2 mixed factors analysis of variance (within/between subjects) was conducted with the inclusion of gender as a covariate (ANCOVA).

### 2.3 Ethical approval

All procedures performed in this study were in accordance with the ethical standards of the Italian Association of Psychology (AIP) research committee and with the 1964 Helsinki declaration and its later amendments.

## 3 RESULTS

The students that were eligible for participation in the present study made a total sample size of 306 students. Therefore, the sample size used in the analysis included 194 students in the information session on stress management (ISSM) alone group condition and 112 students in the ISSM + BWM group condition (306 students in total). The sample was comprised of first‐year university students. Baseline demographics of the sample are shown in Table 1 and suggest that the sample was mostly made up of white individuals, with slightly more males than females.

**TABLE 1:** Demographics of participants by group

	ISSM alone condition	ISSM + BWM condition
*N*	194	112
Age	*M* = 20.59; *SD* = 0.84	*M* = 20,45; *SD* = 0.71
Gender		
Female	*n* = 64 (33.0%)	*n* = 68 (60.7%)
Male	*n* = 130 (67.0%)	*n* = 44 (39.3%)
Level of education		
First‐year of bachelor's degree	*n* = 194 (100%)	*n* = 112 (100%)
Race		
White	*n* = 190 (98%)	*n* = 111 (99%)
More than one	*n* = 4 (2%)	*n* = 1 (1%)

**FIGURE 6:**
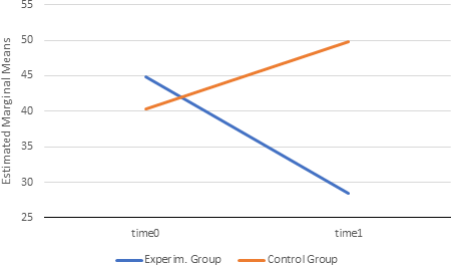
Results of the analysis of variance with the inclusion of gender as a covariate (ANCOVA)

Results of the analysis of variance with the inclusion of gender as a covariate (ANCOVA) showed that there was a statistically significant interaction between time and group (*F*1, 303 = 56.825; *p* < .001; *η* ^2^p = 0.16), as shown in Figure 6. Results also indicate that the intervention did not cause any side effects in the population considered, since none of the participants reported any unusual reactions either postintervention or in the following days.

## 4 DISCUSSION

This study shows the positive effect that the BWM application can have in reducing the perceived level of stress as compared to a single information session on stress management.

The use of the brain wave modulation offers some advantages over a single information session on stress management. Research shows that mind–body techniques, such as bioenergy, biofeedback, mindfulness, cognitive‐behavioral therapy, and yoga, are effective in lowering the students’ levels of stress (Chaló, Pereira, Batista, & Sancho, 2017; Finkelstein‐Fox et al., 2018; Regehr, Glancy, & Pitts, 2013; Running & Hildreth, 2017; Saoji et al., 2017), yet they may present issues limiting their application, in which the stress reduction intervention we used does not present. These limitations include the need for special premises, specific equipment, or body skills. Furthermore, other techniques are generally time‐consuming to perform, and some others require a training that can be challenging to learn. In addition to this, conflicting time demands for multisession schedules are among the other limitations.

Conversely, the BWM is very easy to learn and can be performed in minutes. Students might prefer it over other methods that are more time‐consuming and difficult to learn. Neither special premises nor specific equipment is required. It can be administered individually as well as to a large number of subjects at the same time and is shown to be effective after just one session. Moreover, the BWM only takes one psychologist to administer it to a large number of students, and, once learned, the technique can be performed autonomously at home. Finally, no side effects were reported by any of the participants in this study.

Despite the significant effect found, the present study has several limitations that should be considered when interpreting the results. First, the selection of the control group was not ideal since there was no control for effects such as placebo. Moreover, we measured stress using a single‐item scale, although longer measures for the screening of stress might have been more suited. Yet, we opted for a single‐item scale because of the advantages it offers in terms of brevity and ease of administration. Our choice was based on a body of literature that validated the DT against other robust measures (Snowden et al., 2014) and confirmed that the single‐item DT can be favorably compared with other measures (Jacobsen et al., 2005). Another limitation to the present study is that the impact of the intervention was measured immediately after it was over. In future studies, measurements should be made at longer intervals, including follow‐ups.

Since stress among university students is a widespread and growing problem (Beiter et al., 2015; Chaló et al., 2017; Regehr et al., 2013; Running & Hildreth, 2017), we believe that universities should develop more effective and sustainable stress management programs for students that are based on mind–body therapies (such as mindfulness, yoga, bioenergy, biofeedback, autogenous training, cognitive‐behavioral therapy, and yoga). In particular, it might be worth experimenting innovative methods like the BWM, given the immediate stress reduction effect and the other advantages it presents in its application.

In our view, future studies should investigate the effects of this kind of intervention in order to understand how to maintain the benefits over time and how different students react to the intervention. For this reason, we hope that researchers will further investigate this field of study taking into consideration the possible use of the BWM technique.

## CONFLICT OF INTEREST

The authors whose names are listed immediately below certify that they have NO affiliations with or involvement in any organization or entity with any financial interest (such as honoraria; educational grants; participation in speakers’ bureaus; membership, employment, consultancies, stock ownership, or other equity interest; and expert testimony or patent‐licensing arrangements), or nonfinancial interest (such as personal or professional relationships, affiliations, knowledge, or beliefs) in the subject matter or materials discussed in this manuscript.

## AUTHORS CONTRIBUTION

Mauro Cozzolino and Giovanna Celia developed the theory. Mauro Cozzolino and Laura Girelli carried out the experiment. Laura Girelli performed the computations. Mauro Cozzolino and Deborah R. Vivo wrote the manuscript. Pierpaolo Limone and Giovanna Celia supervised the project.

## Data Availability

The data that support the findings of this study are available from the corresponding author upon reasonable request.

## Appendix 1

### The Administration of the Brain Wave Modulation Technique

Find a relaxing place where no causes of disturbance are present (such as noise or vibrating/ringing phones). Sit on an armchair or a comfortable chair with your legs placed on the ground and the back aligned to the seatback. Put both hands on the legs or on the armrest of the chair and keep eyes closed.

First phase: Be able to touch the extremity of the little finger with the extremity of the thumb (Figure 2).
Second phase: Be able to touch the extremity of the ring finger and the extremity of the thumb (Figure 3).
Third phase: Be able to touch the extremity of the middle finger and the extremity of the thumb (Figure 4).
Fourth phase: Be able to touch the extremity of the middle finger and ring finger with the extremity of the thumb (Figure 5).

All the phases must be done with both left and right hand simultaneously. It is important that the extremities touch each other perfectly (Cozzolino & Celia, 2016).
